# Effect of word association on linguistic event-related potentials in moderately to mildly constraining sentences

**DOI:** 10.1038/s41598-018-25723-y

**Published:** 2018-05-08

**Authors:** Elvira Khachatryan, Mansoureh Fahimi Hnazaee, Marc M. Van Hulle

**Affiliations:** Department of Neuroscience, KU Leuven, Leuven, Belgium

## Abstract

The processing of word associations in sentence context depends on several factors. EEG studies have shown that when the expectation of the upcoming word is high (high semantic constraint), the within-sentence word association plays a negligible role, whereas in the opposite case, when there is no expectation (as in pseudo-sentences), the role of word association becomes more pronounced. However, what happens when the expectations are not high (mild to moderate semantic constraint) is not yet clear. By adopting a cross-factorial design, crossing sentence congruity with within-sentence word association, our EEG recordings show that association comes into play during semantic processing of the word only when the sentence is meaningless. We also performed an exploratory source localization analysis of our EEG recordings to chart the brain regions putatively implicated in processing the said factors and showed its complementarity to EEG temporal analysis. This study furthers our knowledge on sentence processing and the brain networks involved in it.

## Introduction

Comprehension of language (written or spoken) is an intricate process that calls upon a network of brain regions predominantly located in the temporal and frontal lobes, limbic system, parietal and occipital cortices^1^. Since the sentence context develops over time, the processing of a word here differs from that of an isolated word or the one paired with another word^2,3^. The former is being influenced by a number of additional factors, such as the position of the word in the sentence^3^, the expectation of the upcoming word^4^, the semantic constraint imposed by the presented context (sentence)^5^ and so on.

Computational models^6,7^ suggest that sentence formation is the outcome of a combination of word associations and expectations of the upcoming word. The role of word associations in sentence comprehension has been previously studied^8–11^, but the results of neuropsychological investigations are not always in accordance, and the contribution of word association in sentence comprehension remains understudied.

One of the ways to study this question is with event-related potentials (ERPs)^12^, as it provides the temporal resolution needed to investigate the processing of individual linguistic components. Specifically, the N400 potential^13^ has been widely used in linguistic studies. It is a negative going EEG potential that peaks around 400 ms post-onset of the stimulus of interest (‘target’) and reflects the processing of a potentially meaningful stimulus (both linguistic and non-linguistic). When recorded in a word-pair paradigm, the N400 amplitude increases in response to a target that is unrelated to the preceding word (‘prime’, semantic priming)^14^. In a sentence paradigm, the N400 amplitude increases in response to semantic violation and to a drop in word expectancy^4,15^. Another ERP component often observed in linguistic studies is the P600, a positive component, peaking around 600 ms post-onset. Its significance covers a wide range of functions, including the integration of a word in the context^16^, the re-analysis of the received information in well-formed sentences (garden-path effect) and in syntactic violations^17^.

Only few ERP studies investigated the effect of word association in sentence context. For instance, Van Petten *et al*.^11^ and Coulson *et al*.^9^ showed that the N400 amplitude in response to the final word in a sentence with high semantic constraint (when the expectations of the upcoming word were higher than 70%) was unaffected by intra-sentence word association. Instead, the effect of word association was detected in a later time window, as an increased P600 amplitude was observed in response to meaningless sentences with associations present. This observation was explained as a failed attempt to re-analyze and comprehend these sentences.

In another group of ERP studies^8,10,18^ it was shown that word association exhibits -to some degree-, an effect, when the context provided by the sentence is distorted or insufficient to develop an expectation about the upcoming word. For instance, Van Petten *et al*.^10,18^ embedded associated and non-associated word-pairs such as ‘*moon – star’* and ‘*week – species’* in meaningful sentences or pseudo-sentences (grammatically correct combination of words that does not carry any meaning) and showed that word-associations and sentence-level information both play a role in the formation of the N400 amplitude. Furthermore, those effects can be additive. In their follow-up study, Coulson *et al*.^8^ embedded associated and unassociated word pairs in meaningful and meaningless sentences with high semantic constraint and presented them to only one visual hemi-field. They showed that, in this case, word association modulates the N400 amplitude in response to both meaningful and meaningless sentences, but this effect depended on the visual hemifield in which the stimuli were presented. In all studies, where the effect of word-association on N400 in sentence processing was present, it was always surpassed by the effect of sentence meaning. As to the P600, in both Van Petten’s studies^10,18^ only the effect of sentence meaning on P600 amplitude was present but no effect of word-association. For Coulson *et al*.^8^, on the other hand, the effect of word-association in this time-window was significant only for incongruent sentences and when presenting them to the right visual field.

There are even fewer reports on the effect of lexical association on N400 or/and P600 amplitude in sentences with low semantic constraint^19,20^. In these studies, however, the authors manipulated either subject animacy or grammatical congruity. For instance, Kuperberg *et al*.^19^ replaced the animated subject (as in the sentence “During the *breakfast*, the **boys** would eat…”) with the inanimate subject related to the previous context (i.e., “During the *breakfast*, the **eggs** would *eat*…..”). Furthermore, Hoeks *et al*.^20^ replaced the auxiliary verb in the passive sentence like “The *javelin* was by the *athletes* **thrown**.” with the one that rendered the whole sentence incongruent (i.e., “The *javelin* has by the *athlete* **thrown**.”). In both studies, the N400 amplitude in response to the incongruent sentences with the lexical associations (egg – eat and javelin – athlete – thrown) was decreased, but instead, these sentences evoked a significantly larger P600. This phenomenon was called “semantic illusion”; that is, the subject’s preferences to accept these sentences as semantically correct and “notice” the violation only during re-evaluation (P600 – re-analysis). However, here, besides semantic violation, one can also observe a syntactic manipulation, which renders these sentences syntactically ambiguous.

In all mentioned studies, either sentences with strong semantic constraint (no effect), pseudo-sentences or syntactically ambiguous sentences (significant word-association effect) were tested.

Therefore, it is still not clear whether the lexical association effect can be present in the semantically low constraining but still valid sentences without syntactic ambiguity.

We are aware of only one ERP study^21^ that investigated the effect of word-association in discourses with low to moderate semantic constraint. Using both eye-tracking and ERP techniques, they showed that the effect of word association can be elicited in the processing of discourses, when congruity of the evaluated discourse is interrupted. Here, unlike previous studies, they always used congruent sentences and the plausibility of the discourse was manipulated via the preceding context (two sentences). When they additionally studied the role of word associations in a context of a single sentence (by using eye tracking, cf. their experiment 4), they did not take into account the cloze probabilities of those sentences, which raises a question of its possible effect on the processing of the target word.

Some time ago, we investigated the effect of word-association in moderately to mildly constraining sentences (average cloze probability around 50% for each sentence group) on the N400 amplitude in a pilot study testing three subjects using 32 EEG electrodes^22^. Here, we adopted a semantic anomaly judgement task, that is, the subjects had to decide on the semantic congruity of the sentences. We observed no significant effect of word association during N400 time window. In contrary, during this time window, a considerable effect of sentence congruity was observed. The P600 time window was not considered in this study. In the current study, we assess the validity of our preliminary findings by testing more subjects and employing a high-density EEG (128 electrodes), as well as verify the effect of word-association in the processing of mildly to moderately constraining valid unambiguous sentences. In addition, instead of considering an explicit semantic anomaly judgement task^22^ or a reading on comprehension (and possible rephrasing) task, as in most previous studies^8,21^, we used a lexical matching (probe verification) task to avoid the reader’s bias towards the meaning of the sentences.

If there is any effect of word association in the processing of mild to moderately constraining sentences, the considered task is perfect for revealing it, since this task is independent from sentence meaning and the presence of associations. The presence of the said effect will be manifested with the difference between N400 and/or P600 amplitude in response to sentences with and without associations in addition to or independent from the effect of sentence congruity.

Besides scalp-based ERP analysis, our high-density EEG recordings allowed us to explore the neural sources of the targeted effects on individual ERPs with an accuracy of centimeters. Neural generators of the N400 have been identified in several regions, including the left temporal cortex, left and right lateral prefrontal cortex, left angular gyrus, right temporal cortex, and both anterior and posterior cingulate gyri for incongruent context of a sentence^23^. Other studies showed the neural generators of the N400 effect (the difference between N400 amplitudes for the group of interest and the reference group) to be related to the activation of occipitotemporal and parahippocampal gyri, and anterior temporal lobes bilaterally^24,25^. Intracranial studies using sentential stimuli with incongruent endings observed an ERP with characteristics similar to the N400 to be located bilaterally in the anterior temporal lobe^25^. Interestingly, even though the scalp distribution of N400 has been shown to be larger at lateral sites of the right hemisphere^13^, studies with split-brain patients indicate the presence of left-hemisphere generators for the N400 effect^26^.

Similar to the N400, a broad range of brain areas are activated in the P600 time-window, among which we note the left middle temporal gyrus, the posterior part of the temporal lobe and the left inferior frontal gyrus^16^. Other studies suggest activation in the anterior portion of the left superior temporal gyrus and left fronto-opercular cortex. For instance, Friederici *et al*.^27^ speculated that basal ganglia structures, in particular, the caudate nucleus, the putamen and the globus pallidus play an important role in the controlled syntactic processes of the P600. In our study, we additionally performed source localization to assess how the effects of sentence congruity and intra-sentence word-associations correlate with known cerebral generators of N400 and P600 for the case of mild to moderately constraining sentences. Considering previous studies that made claims on the inter-hemispheric differences between sentence versus lexical information processing^8,28^ (albeit they used sentences with high semantic constraint), we additionally hypothesize that the neural generators of the N400 and P600 respond differently depending on the congruity of the sentence and the presence of intra-sentence associations.

## Methods

### Participants

A group of 16 healthy young graduate and undergraduate students [average age 23.3 years old, ranging between 19 and 26 years, 7 females, 2 left handed] participated in the study. All participants had Dutch as their mother tongue. Participants were paid volunteers. All subjects had normal or corrected to normal vision, none of them reported a history of any neurological or psychiatric condition. The study was conducted in accordance with the latest version (2013) of the Declaration of Helsinki, following ethical approval obtained from Leuven University Hospitals’ Ethical Committee. After being informed about the set-up and goal of the experiment, all subjects gave their written informed consent.

### Materials

The development of the stimuli list (see Supplementary Material, Appendix 2) started with the creation of 80 Flemish-Dutch declarative sentence stems (all words in the sentence, besides the last - ‘target’ word). Afterwards, the last word of each sentence was obtained by testing 40 undergraduate and graduate students of KU Leuven on a cloze probability task, during which participants needed to finish the sentence stem with the word that first came to mind. The cloze probabilities (the percentage of the participants that responded with that particular word to that particular sentence stem) for the best matches of all sentences ranged between 16 and 68% (the average 46%). Therefore, these sentences had a mild to moderate semantic constraint. All sentences were active and always ended with a noun. In 40, out of the originally 80 sentences, the target word was lexically associated with one of the previous words in the sentence called ‘prime’ word. Since, the experiment was designed by crossing the factors of sentence congruity and lexical associations between target and prime words, 80 meaningless sentences were created from the original meaningful sentences by changing the target word with the one that rendered the sentence meaningless. Similar to the meaningful ones, in 40 out of the 80 meaningless sentences, the lexical association between prime and target words was present. To define the prime word of a sentence, the association strength (AS) value between the target word and each word of the sentence stem was calculated using the word association database developed at the Psychology Department of KU Leuven^29^. The word with the highest AS value of a given sentence was considered as prime of that sentence. Hence, to calculate the average AS value per stimulus group, we took the average of the highest AS values of each sentence in that particular group. For the meaningful sentences with lexical associations, the average AS value was around 0.051 (std = 0.053), while for the meaningless sentences with association it was 0.063 (std = 0.054). For meaningful sentences without association the average AS value was 0.0027 (std = 0.0054) and for meaningless sentences without association, it was around 0.0008 (std = 0.003). The repeated measure ANOVA with sentence congruity (SC), word-association (WA) and their SC x WA interaction as independent factors, and AS values per sentence as dependent variables showed a significant effect of WA (F(1, 156) = 78.36, p < 0.0001). The effects of neither SC (p = 0.43), nor SC x WA interaction (p = 0.26) were significant. Additionally, the measures were taken to avoid the orthographic overlap between prime and target words. For that, we calculated the Levenshtein distance for orthographic similarity^30^ between prime and target words for each sentence. The average minimal Levenshtein distance across sentences was 3.8 (std = 1.36) and the repeated measure ANOVA did not show a significant difference between the values across sentence groups (p = 0.17), therefore, the observed effects can be reliably attributed to the associative/semantic relatedness between prime and target words rather than to their orthographic similarity. Eventually, we developed four groups of sentences: congruent with and without associations and incongruent with and without associations (Table 1 for sentence groups and exemplar sentences, also note our abbreviations of each sentence group for quick referencing). Repeated measure ANOVA did not reveal any significant difference between cloze probabilities of the four sentence groups (p = 0.23).

**Table 1:** Sentence groups, exemplar sentences and their literal translations into English.

Sentence group	Exemplar sentences	Literal translation of the sentence
Congruent-associated (congHA)	De *zakenman* droeg een mooi **pak**.	The *businessman* wore nice **suit**.
Congruent-unassociated (congLA)	Tom erfde *grond* van zijn **vader**.	Tom inherited the *land* from his **father**.
Incongruent-associated (incongHA)	Tom erfde *grond* van zijn **aarde**	Tom inherited the *land* from his **earth**.
Incongruent-unassociated (incongLA)	De *zakenman* droeg een mooi **doek**.	The *businessman* wore nice **canvas**.

Prime words are in italic, whereas target words – in bold.

The frequency of the target words for all sentence groups were checked using Dutch SUBTLEX word frequency database^31^. Word length and orthographic neighborhood size (number of words that can be generated if one letter in the word is changed) of target words were obtained via CLEARPOND publicly available software^32^. Repeated measure ANOVA showed no statistically significant difference between targets of all four sentence groups on any of the mentioned characteristics: word frequency p = 0.185, word length p = 0.13, and orthographic neighborhood size p = 0.8.

Finally, in order to reassure ourselves that the level of congruity did not depend on associations between word pairs in the sentences, we split our stimuli (160 sentences, both congruent and incongruent) into two lists and showed them to 8 colleagues, who were blind to the sentence group for their judgment of the congruity level. Each person saw only one version of the sentence (congruent or incongruent), thus they did not have a chance to compare congruity level of different versions of the same sentence. Hence, each sentence was judged by 4 participants. They were judging the sentence using a 7-point scale with 1 absolutely meaningless to 7 making perfect sense. Congruent associated and unassociated sentences had scores of 6.8 and 6.9 respectively, while incongruent associated and unassociated sentences had scores of 2 and 1.67 respectively.

### Experimental procedure

Subjects were tested in a sound-attenuated experimental room sitting on a chair in front of an LCD screen. The sentence stem appeared on the screen at once with white letters on a black background during 2.5 s, which was deemed sufficient for the subject to read the sentence stem. Then, a fixation cross appeared in the center of the screen, prompting the subject to keep his/her eyes fixated on the position where the target word will appear. The fixation cross remained on the screen during 300 ms, followed by the target word during 500 ms. Each subject saw each target word only once. The presentation of the sentence stems was counterbalanced in a way that half of the stems was presented first in the meaningful context while the other half was shown first in the meaningless context.

After the presentation of the target word, the blank screen appeared for 700 ms, which was followed by a screen with a probe word in capitals with two options: “Ja” (yes) or “Nee” (no). The subject needed to respond by button-press if he/she saw this word in the previously presented sentence (the whole sentence, thus, including the target word). The positive response had to be given by pressing the left mouse button, the negative one by pressing the right button. After the response, the feedback appeared on the screen for 500 ms, which did not reflect the correctness of the subject’s answer, but rather was a reminder about the role of each button. The response hand was counterbalanced across subjects: half of the subjects were responding with their left hand, the other half with their right hand.

Prior to the main experiment, each subject completed a short training session consisting of five sentences. The entire experiment was split into shorter blocks, thus, subjects could take breaks every 5–7 minutes. During each block, sentences from all 4 groups were shown to the subjects in the pseudo-random way.

### EEG acquisition

The EEG signal was acquired using high-density 128 active Ag/AgCl electrodes mounted in a cap placed on the subject’s head according to the international 10–5 system. Conductive gel was applied into the halls of the electrode cap in order to improve impedance between the electrodes and the subject’s skin. The impedance was kept below 5 kΩ. The signal was acquired continuously and amplified with a Synamps RT device (Compumedics, Australia) at a 2 kHz sampling rate. The whole experiment with the cap mounting and the breaks lasted approximately 1 hour.

### Data analysis

The recorded signal from each electrode was re-referenced offline from the original reference (FCz) channel to an averaged mastoid reference and filtered twice using a fourth-order Butterworth filter: low-pass filtered with cutoff frequency of 15 Hz, and high-pass filtered with a cutoff frequency of 0.5 Hz. Then, the signal was cut into epochs starting 300 ms prior to the onset of the target word till 1000 ms post-onset. For the temporal analysis, we selected 31 electrodes distributed evenly over the scalp (AF3, AF4, F7, F3, Fz, F4, F8, FC5, FC1, FC2, FC6, T7, C3, Cz, C4, T8, CP5, CP1, CP2, CP6, P7, P3, Pz, P4, P8, PO3, POz, PO4 and O1, Oz and O2). In order to clean the signal from possible artifacts, we set the threshold on 50 μV, and removed all epochs that exceeded the set threshold at any moment in time on any of the selected electrodes. For the remaining trials, the baseline was removed using the average of EEG signal from 300 ms pre-onset of the target word till the onset (0 ms). As the N400 and P600 amplitudes, we took the average EEG amplitudes (area under the curve) in the 300–500 ms and 500–800 ms intervals, respectively.

### Source localization

Source localization was performed with the Brainstorm toolbox^33^, documented and freely available under the GNU general public license. As our head model, we used the ICBM152 template as the default template, an average of 152 subjects. The forward model used to compute the head model was OpenMEEG BEM^34^, for which we took 15000 dipoles uniformly distributed over the cortical surface. For each subject separately, a subset of bad channels (between 2 and 11 channels, avr 5.2) with largest amplitudes were selected and eliminated from source analysis. Additionally from the remaining 110 channels per subject, we discarded the epochs that still exceeded the 150 μV threshold. This threshold was taken higher than the threshold of ERP analysis (see, section Data analysis), as source analysis requires as many electrodes and trials as possible and lower thresholds would result in loss of a significant number of trials. For the remaining epochs, the noise covariance matrix was obtained by merging the matrices calculated from the baseline of all remaining trials. As to our inverse methods, we used the sLORETA algorithm^35^, which suggested to yield zero localization error. The source orientations where specified to be constrained, which means they are constrained to be perpendicular to the cortical surface. Other parameters required for sLORETA where left to be the default parameters provided and justified by the Brainstorm Toolbox.

### Statistical analysis

For the behavioral data, repeated measure ANOVA was used with the sentence congruity (SC, 2 levels: congruent, incongruent), word association (WA, 2 levels: associated, non-associated), the presence of the probe word in the sentence (PW, 2 levels: present, absent) and their interaction (SC × WA × PW, 2 × 2 × 2 design) as independent effects and each subject’s response accuracy for each sentence group and each type of probe word as dependent variables.

For the EEG data, mixed design ANOVA^36^ was used with SC, WA and their SC × WA interaction (2 × 2 design) as fixed effects, subject as random effect and amplitudes of N400 and P600 as dependent variables for each selected electrode. Multiple comparison was performed using Student’s t-test with false discovery rate (FDR) correction where appropriate. Test results with p-value below 0.05 after correction on multiple comparison were considered to be significant and are presented across the manuscript.

For source space data, again, mixed design ANOVA was used with the same setting except that this time, as dependent variables dipole amplitude over an average of 300–500 ms and 500–800 ms were used for N400 and P600 analysis, respectively. This was done on each of the investigated 15000 dipoles on the cortical surface. The p-value for each dipole was corrected using Bonferroni correction method.

### Data Availability

The anonymized and pre-processed data that supports the findings of this study are available from corresponding author upon request.

## Results

### Behavioral data

The average performance on all sentence groups was 0.9. The performance on individual sentence groups (standard deviation between brackets) was as follows: for congruent-associated 0.88 (std = 0.021), for congruent-unassociated 0.86 (std = 0.03), for both incongruent-associated and incongruent-unassociated groups 0.94 (std = 0.04 and 0.03, respectively). Repeated measure ANOVA with the factors of SC, WA and PW (three-way interactive model) showed a significant effect of SC (F(1, 120) = 27.16, p < 0.0005), PW (F(1, 120) = 13.88, p < 0.0005) and their SC × PW interaction (F(1, 120) = 18.4, p < 0.0005). No other effects were significant. The further investigation of performance on each sentence group with and without presence of probe word in the sentence showed a lower performance for the congruent sentences, when the probe word was not present in the sentence: 0.835 versus 0.94 for all other conditions.

### ERP data

**Figure 1:**
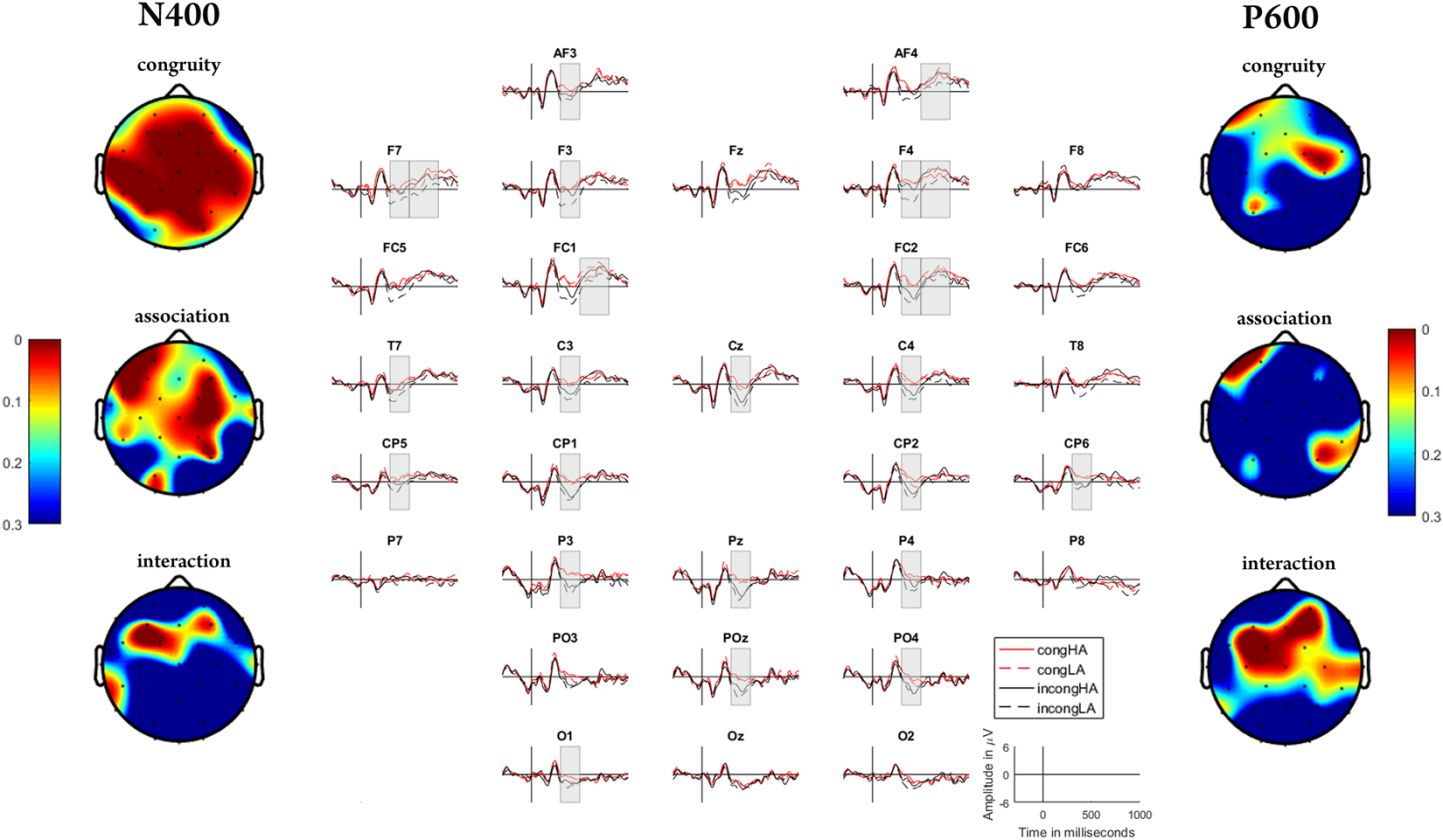
Grand average ERPs of all 16 subjects tested, plotted for the four studied sentence groups and the spatial distribution of the considered effects.

For the **N400 time window (**Fig. 1**)**, mixed design ANOVA with SC, WA and their interaction (2 × 2 design) as fixed effects and subject as random effect showed a significant effect of sentence meaning in mainly centrally localized electrodes (for electrode Cz, F = 14.64, p = 0.0017). The effect of association was more present on the right hemisphere (for electrode C4, F = 7.49, p = 0.015). The effect of SC × WA interaction was significant on very few electrodes that were more frontally located (for electrode F3, F = 7.63, p = 0.0058).

A further pairwise comparison using Student’s t-test with FDR correction showed that both incongruent (incongHA and incongLA) sentence groups had more negative N400 amplitude compared to both congruent (congHA and congLA) sentence groups (in all cases, for electrode Cz, p < 0.05). Here, the incongruent sentence group with associations (incongHA) showed significantly less negativity compared to the incongruent sentence group without associations (incongLA) (for electrode Cz, t = 2.32, p = 0.02). This pattern of ERPs was more centrally located. For the other electrode locations, the main difference was for the congruent sentence groups from the incongLA group (all cases, p < 0.05). In these locations, the incongHA group was not significantly different from either one of the other three groups. An exploratory source localization analysis (Supplementary Material, Appendix 1) in this time window revealed a large effect of congruity mainly in the basal temporal cortex (Table S1, Supplementary Material). The effect of association was considerably smaller and mainly located in the frontal cortex (mainly of the right hemisphere) (Fig. S2, Supplementary Material).

For the **P600 time-window**, mixed design ANOVA with the abovementioned factors showed significance mainly for SC× WA interaction (for electrode Cz, F = 6.92, p = 0.0086). A further pairwise comparison showed a significant difference mainly between the two groups without associations (congLA versus incongLA) (for electrode Cz, t = 2.92, p = 0.0035) with a more positive P600 in response to congLA group. Source localization analysis showed an effect of congruity in the left basal temporal cortex (Table S2, Supplementary Material) but only for unassociated sentences. The effect of association similar to the N400 time-window, was present in the frontal cortex and again mainly in the right hemisphere (Figs S1 and S2, Table S2, Supplementary Material).

## Discussion

We investigated the effect of word association in processing congruent and incongruent sentences from the perspective of ERP analysis and source localization. Here, the sentences were legal, therefore, evoking expectations for every upcoming word, but had mild to moderate semantic constraint, implying that those expectations were not high. Based on our ERP analysis, we showed that the effect of word association in sentence context during retrieval of word semantics^16^, comes into play only when the congruity of the context is disrupted, as we observed this effect in incongruent sentences only.

As reading for comprehension would bias the subject towards the factor of congruity, in order to avoid such bias, we designed the experiment so, as to ensure that each word was fully processed, but at the same time would not concentrate on sentence congruity or the possible presence of within sentence associations.

Here, the results indicate that the semantic retrieval of the final word expressed with N400 in the incongruent sentences with associations was facilitated compared to the processing of the same word in incongruent sentences without associations. This observation was also present in previous studies with paradigms containing intra-sentential word associations^8,10,18^, but for instance, Coulson *et al*.^8^ with their mono-hemispheric presentation of the stimulus were more interested in the investigation of differences in hemispheric processing rather than in the role of word association in the sentence context. At the same time, Van Petten *et al*.^10,18^ used pseudo-sentences instead of legal sentences, rendering the generation of the prediction in their incongruent sentences impossible. Unlike them, we concentrated on the processing of legal sentences with mild semantic constraint and the role of word association in semantic processing of words embedded in sentences when there are mild expectations about the upcoming word. We showed that this effect becomes visible only in the case of a meaningless context. Unlike the study conducted by Camblin *et al*.^21^, who were evaluating the effect of lexical associations in the processing of single sentences, we did not observe a difference between the two congruent sentence groups (with and without associations). Since Camblin *et al*.^21^ did not correct their individual sentences for a possible influence of cloze probability; we suggest that the observed effect in their study might simply reflect the difference in cloze probabilities between associated and unassociated groups.

The results from the late time window (P600) showed a significant effect of SC × WA interaction with a significantly larger positivity in response to congruent sentences without associations (congLA) compared to the incongruent sentences without associations (incongLA). This ERP, originally believed to represent the syntactic processing in the sentence context^17^, is currently suggested to reflect more cognitive processes, such as processing of semantics^37^ and pragmatics^19^, as well as word integration^16^ in the sentence. In case of so-called ‘semantic illusion’, the P600 amplitude increases in response to semantic or pragmatic violation, while N400 amplitude remains the same. All these studies suggest that the modulation of P600 in response to experimental manipulation is a result of re-analysis and a failed attempt to fit the read word in the previous context. In the current study, the significant effect of SC × WA interaction observed during P600 time-window can be similarly interpreted. However, noteworthy, unlike previous studies where larger P600 amplitude was observed in response to incongruent sentences with associations compared to the other sentences^19,20^, in our case, a larger positivity in P600 time-window was observed in response to meaningful sentences without associations compared to the meaningless sentences without associations (Fig. 1). If similar to computational models of sentence processing^6,7^, we assume that the comprehension of a single word in a sentence context is a result of combined expectation generation and intra-sentential word-association, this pattern during P600 time window can be explained by additional effort spent on the processing of meaningful sentences without associations. Indeed, since the issue of meaningfulness was solved already during the time-window of N400 (a significant effect of sentence congruity in this time window), further re-analysis and integration occurring during P600 time-window would be directed on the optimization of the word-processing in a sentence context. A similar effect was observed by Van Petten^10,18^. This suggests that the influence of lexical association on word processing in sentence context during word integration^16^ and re-analysis might be even stronger compared to the earlier processes of semantic retrieval.

Interestingly, our current results differ from the previous ones where we used a similar stimuli list^22^. In Khachatryan *et al*.^22^, using mildly to moderately constraining sentences with embedded associated and unassociated word-pairs and explicit semantic anomaly judgement task, similar to Van Petten *et al*.^11^, we did not observe any effect of word association in any of the contexts (congruent or not), from which, we concluded that if the sentence evokes any, even mild expectations about the upcoming word and the sentences are syntactically unambiguous; the processing of this word will fully be driven by these expectations rather than the presence of lexical associations. We hypothesize that this discrepancy in outcomes could be due to the difference in tasks that were employed. It was shown that the task has a significant influence on the amplitudes of both N400^38^ and P600^39^, therefore, it is possible that the change in task can influence the ERP pattern we see in our study. The explicit semantic judgement anomaly task employed in Khachatryan *et al*.^22^ evokes an N400 with larger amplitude and eases its evaluation. However, it causes the subject to pay more attention to sentence congruity and therefore to process the sentence and each word more based on its integrity with the preceding context as a whole. In the current study, we employed a lexical matching task (probe verification), which enabled us to detect an (although small) effect of lower level intra-sentential association on word processing in sentence context that was missed by Khachatryan *et al*.^22^, a pilot study with only 3 subjects and therefore to be approached with caution. However, with the current comparison we would like to stress the importance of employed task on the obtained results, which is also in line with some previous reports^38,39^.

Furthermore, as we were using high-density EEG, we managed to perform an exploratory source localization analysis and obtain information about possible generators of the observed effects on the amplitudes of our ERPs in response to different sentence groups. We observed a significant effect of congruity in the basal temporal cortex (including fusiform gyrus and parahippocampal cortex), which was previously suggested^25,40^ to participate in language processing. This effect, unlike the one in Nobre & McCarthy^41^, was independent from word association. We hypothesize that this could be due to the semantic priming resulting from intra-sentential lexical associations being more of an automatic nature, while sentence congruity covers the controlled part of sentence processing. As the basal temporal cortex probably reflects controlled language processing, the effect of word association was not observed here. Furthermore, the left supramarginal gyrus also showed a significant effect of congruity in the early time-window (Table S1, Supplementary Material). This region is also known for its involvement in language processing, among other functions^1,42^.

The effect of word association in general was more pronounced in the frontal cortex and in the right hemisphere in particular, while the effect of congruity was spread on both hemispheres (Supplementary Material). This suggests that right hemisphere in addition to the processing of the meaning via a controlled mechanisms, participates in basic lexical processing. This observation is in accordance with previous mono-hemispheric studies of lexical and semantic processing^43^ and with the recent intracranial study that employed similar cross-factorial design^44^.

## Conclusion

With the current study, we extended our knowledge about the effect of word association in sentence context. We advocate that when processing mildly constraining sentences, the brain additionally relies on word associations. When sentence congruity is impaired and a neutral task is employed, the effect of word-association can be observed during semantic processing of the word expressed in terms of N400. Later, during P600 time-window, when the re-analysis of the received information takes place, the role of an intra-sentential word-association in the processing of the target word in the sentence increases. We showed this effect for syntactically unambiguous sentences that have mild to moderate expectations for every upcoming word. In addition, we showed that the right hemisphere was more involved in the processing of within sentence word associations compared to the left hemisphere, advocating the unique contribution of each hemisphere in the processing of a word in a sentence.

## Electronic supplementary material

Supplementary material

## References

[1] Lau EF, Phillips C, Poeppel D (2008). A cortical network for semantics: (de)constructing the N400. Nat. Rev. Neurosci..

[2] Borovsky A, Elman JL, Kutas M (2012). Word Meanings from a Single Exposure in Context. Lang. Learn. Dev..

[3] Van Petten C, Kutas M (1990). Interactions between sentence context and word frequency in event-related brain potentials. Mem. Cognit..

[4] Kutas M, Hillyard S (1984). Brain potentials during reading reflect word expectancy and semantic association. Nature.

[5] Fischler IRAS, Bloom PA (1985). Effects of constraint and validity of sentence contexts on lexical decisions. Mem. Cognit..

[6] Tabor W, Tanenhaus MK (1999). Dynamical Models of Sentence Processing. Cogn. Sci..

[7] Demberg, V. & Keller, F. A Computational Model of Prediction in Human Parsing: Unifying Locality and Surprisal Effects. In *CogSci 2009 Proceedings*. *Cognitive Science Society* 1888–1893 (2009).

[8] Coulson S, Federmeier KD, Van Petten C, Kutas M (2005). Right hemisphere sensitivity to word- and sentence-level context: evidence from event-related brain potentials. J. Exp. Psychol. Learn. Mem. Cogn..

[9] Coulson, S. *et al*. Lexical and Sentential Context Effects: An ERP study of the difference between life and death and life in prison. in *The Brain Science Connection MITCogNet* (2000).

[10] Van Petten C (1993). A comparison of lexical and sentence-level context effects in event-related potentials. Lang. Cogn. Process..

[11] Van Petten, C. *et al*. Lexical association and higher-level semantic context: An ERP study. *J*. *Cogn*. *Neurosci*. *Suppl*. Supplement, 46 (1999).

[12] Luck, S. J. *An introduction to Event related potential technique*. *Monographs of the Society for Research in Child Development* **79**, (MA: MIT Press, 2005).

[13] Kutas M, Federmeier KD (2011). Thirty years and counting: finding meaning in the N400 component of the event-related brain potential (ERP). Annu. Rev. Psychol..

[14] Holcomb PJ, Neville H (1990). Auditory and Visual Semantic Priming in Lexical Decision: A Comparison Using Event-related Brain Potentials. Lang. Cogn. Process..

[15] Kutas M, Hillyard S (1980). Reading senseless sentences: Brain potentials reflect semantic incongruity. Science (80-.)..

[16] Brouwer H, Hoeks JCJ (2013). A time and place for language comprehension: mapping the N400 and the P600 to a minimal cortical network. Front. Hum. Neurosci..

[17] Gouvea AC, Phillips C, Kazanina N, Poeppel D (2010). The linguistic processes underlying the P600. Lang. Cogn. Process..

[18] Petten, C. V, Weckerly, J., Mclsaac, H. K. & Kutas, M. Working Memory Capacity Dissociates Lexical and Sentential Context Effects. *Psychological science* **8**, (1997).

[19] Kuperberg GR, Sitnikova T, Caplan D, Holcomb PJ (2003). Electrophysiological distinctions in processing conceptual relationships within simple sentences. Cogn. Brain Res..

[20] Hoeks JCJ, Stowe LA, Doedens G (2004). Seeing words in context: The interaction of lexical and sentence level information during reading. Cogn. Brain Res..

[21] Camblin CC, Gordon PC, Swaab TY (2007). The interplay of discourse congruence and lexical association during sentence processing: Evidence from ERPs and eye tracking. J. Mem. Lang..

[22] Khachatryan, E. *et al*. Amplitude of N400 component unaffected by lexical priming for moderately constraining sentences. in *Proceedings of 2014 4th International Workshop on Cognitive Information Processing* 0–5 (2014).

[23] Frishkoff GA, Tucker DM, Davey C, Scherg M (2004). Frontal and posterior sources of event-related potentials in semantic comprehension. Cogn. Brain Res..

[24] Silva-pereyra J, Rivera-gaxiola M, Aubert E (2003). N400 during lexical decision tasks: a current source localization study. Clin. Neurophysiol..

[25] McCarthy G, Nobre AC, Bentin S, Spencer DD (1995). Language-related field potentials in the anterior-medial temporal lobe: I. Intracranial distribution and neural generators. J. Neurosci..

[26] KUTAS M, Hillyard SA, Gazzaniga MS (1988). Processing of semantic anomaly by right and left hemispheres of commissurotomy patients. Brain.

[27] Friederici AD, Rüschemeyer S, Hahne A, Fiebach CJ (2003). The Role of Left Inferior Frontal and Superior Temporal Cortex in Sentence Comprehension: Localizing Syntactic and Semantic Processes. Cereb. Cortex.

[28] Faust M, Babkoff H, Kravetz S (1995). Linguistic processes in the two cerebral hemispheres: implications for modularity vs interactionism. J Clin Exp Neuropsychol..

[29] De Deyne S, Storms G (2008). Word associations: Network and semantic properties. Behav. Res. Methods.

[30] Yarkoni T, Balota D, Yap M (2008). Moving beyond Coltheart’s N: A new measure of orthographic similarity. Psychon. Bull. Rev..

[31] Keuleers E, Brysbaert M, New B (2010). SUBTLEX-NL: a new measure for Dutch word frequency based on film subtitles. Behav. Res. Methods.

[32] Marian V, Bartolotti J, Chabal S, Shook A (2012). CLEARPOND: cross-linguistic easy-access resource for phonological and orthographic neighborhood densities. PLoS One.

[33] Tadel, F., Baillet, S., Mosher, J. C., Pantazis, D. & Leahy, R. M. Brainstorm: A user-friendly application for MEG/EEGanalysis. *Comput*. *Intell*. *Neurosci*. **2011** (2011). 10.1155/2011/879716 PMC3090754 21584256

[34] Gramfort, A., Papadopoulo, T., Olivi, E. & Clerc, M. OpenMEEG: opensource software for quasistatic bioelectromagnetics. *Biomed*. *Eng*. *Online* **9**, (2010). 10.1186/1475-925X-9-45 PMC2949879 20819204

[35] Pascual-Marqui RD (2002). Standardized low-resolution brain electromagnetic tomography (sLORETA): technical details. Methods Find. Exp. Clin. Pharmacol..

[36] Verbeke, G. & Molenberghs, G. *Linear Mixed Models for Longitudinal Data*. (Springer, 2000).

[37] Van de Meerendonk N, Indefrey P, Chwilla DJ, Kolk HHJ (2011). Monitoring in language perception: Electrophysiological and hemodynamic responses to spelling violations. Neuroimage.

[38] Deacon D, Breton F, Ritter W, Vaughan HGJ (1991). The Relationship Between N2 and N400: Scalp Distribution, Stimulus Probability, and Task Relevance. Psychophysiology.

[39] Schacht A, Sommer W, Shmuilovich O, Casado Martienz P, Martin-Loeches M (2014). Differential Task Effects on N400 and P600 Elicited by Semantic and Syntactic Violations. PLoS One.

[40] Burnstine TH (1990). Characterization of the basal temporal language area in patients with left temporal lobe epilepsy. Neurology.

[41] Nobre AC, McCarthy G (1995). Language-related field potentials in the anterior-medial temporal lobe: II. Effects of word type and semantic priming. J. Neurosci..

[42] Balconi M, Vitaloni S (2014). N400 Effect When a Semantic Anomaly is Detected in Action Representation. A Source Localization Analysis. J. Clin. Neurophysiol..

[43] Faust M, Barlev A, Chiarell C (2003). Sentence priming effects in the two cerebral hemispheres: Influences of lexical relatedness, word order, and sentence anomaly. Neuropsychologia.

[44] Khachatryan, E. *et al*. A New Insight into Sentence Comprehension: the Impact of Word Associations in Sentence Processing as Shown by Invasive EEG Recording. *Neuropsychologia* in press (2017). 10.1016/j.neuropsychologia.2017.12.002 29203203

