# Supplementary material for: Effect of word association on linguistic event-related potentials in moderately to mildly constraining sentences
Source: Sci Rep. 2018 May 8;8:7175. doi: 10.1038/s41598-018-25723-y (PMC5940757; doi:10.1038/s41598-018-25723-y)
Supplement: Supplementary file 1 — Supplementary material [file 41598_2018_25723_MOESM1_ESM.pdf]

<u>Supplementary material</u>

Title: Effect of word association on linguistic event-related potentials in moderately to mildly constraining sentences

Authors: Elvira Khachatryan, Mansoureh Fahimi Hnazaee, Marc M. Van Hulle

APPENDIX 1: EXPLORATORY SOURCE LOCALIZATION ANALYSIS

Source localization attempts to find an instantaneous solution to how neural sources relate to electric activity recorded on the scalp[1]. It is based on the principles of electromagnetic conductance across different layers of the head. The spatial accuracy of EEG-based source localization might still be inferior to what fMRI is currently able to offer, however, given the superior temporal resolution of EEG and since it provides a direct measurement of neural activity, the importance of source localization has been steadily growing since the first developments nearly two decades ago[2]. Of course, the actual accuracy depends on many factors, including the accuracy of the head model and the algorithm used for solving the inverse solution mapping from scalp recordings to neural sources. For linguistic ERPs, such as N400 and P600 source localization is challenging, since the linguistic paradigms are expected to activate multiple regions in both hemispheres and therefore, a special approach, called distribution source model, is more appropriate in such cases. Here, the solution space is distributed over the entire cortical volume or cortical surface, and constraints on the spatio-temporal distribution of cortical activity are used to obtain the inverse solution mapping.

Due to the signal conductance through various head tissues (cerebrospinal fluid, skull, skin) and the putatively different dipole directions, it could very well be that scalp electrodes exhibiting largest activity are not the ones closest to the generators of that activity. Since, the principles of volume conduction are far more complex, the conclusions about the neural generators of ERPs based only on scalp activity should be considered with caution[3].

Results

According to Nunez & Srinivasan[4], the area of cortical activation exceeding approximately 6cm^2 will be recordable on the scalp. Therefore, we only analyzed regions that previously to correction on multiple comparison were exceeding this threshold. Most of the areas mentioned in the current paper before the correction had an average size of 6.48cm^2 and 7.1cm^2 for the N400 and P600 ERPs, respectively. Despite this precaution, we excluded from our results the areas that lost their significance after correction on multiple comparison.

N400 ERP

The *effect of congruity* (Table S1, I) for both sentences with and without associations was present in basal temporal cortex bi-hemispherically (fusiform gyrus and parahippocampal gyrus) (Fig. S1, panels A and C) (e.g., for parahippocampal gyri the size of the area with significance was around 10cm^2 in total for both hemispheres, p<0.005 for both sides). The left supramarginal gyrus presented a significant effect of congruity in sentence without associations (area around 1.89 cm^2, p=0.006).

As to the *effect of association (Table S1, II)*, we mainly observed it for the congruent sentences (Fig. S1, C) in the right inferior frontal cortex (area=3.6cm^2, p=0.0044) and frontal pole on both hemispheres (area = 3.45 cm^2, p=0.007).

Table S1: Effect of congruity and association for N400 ERP.

	I. Congruity effect		II. Association effect
**Associated (HA)**	- Right parahippocampal gyrus<br>- Right fusiform gyrus	**Congruent**	- Right inferior frontal cortex<br>- Frontal poles in both hemispheres
**Unassociated (LA)**	- Parahippocampal gyrus on both hemispheres<br>- Left Fusiform gyrus<br>- Left supramarginal gyrus	**Incongruent**	- Left inferior frontal cortex (small)

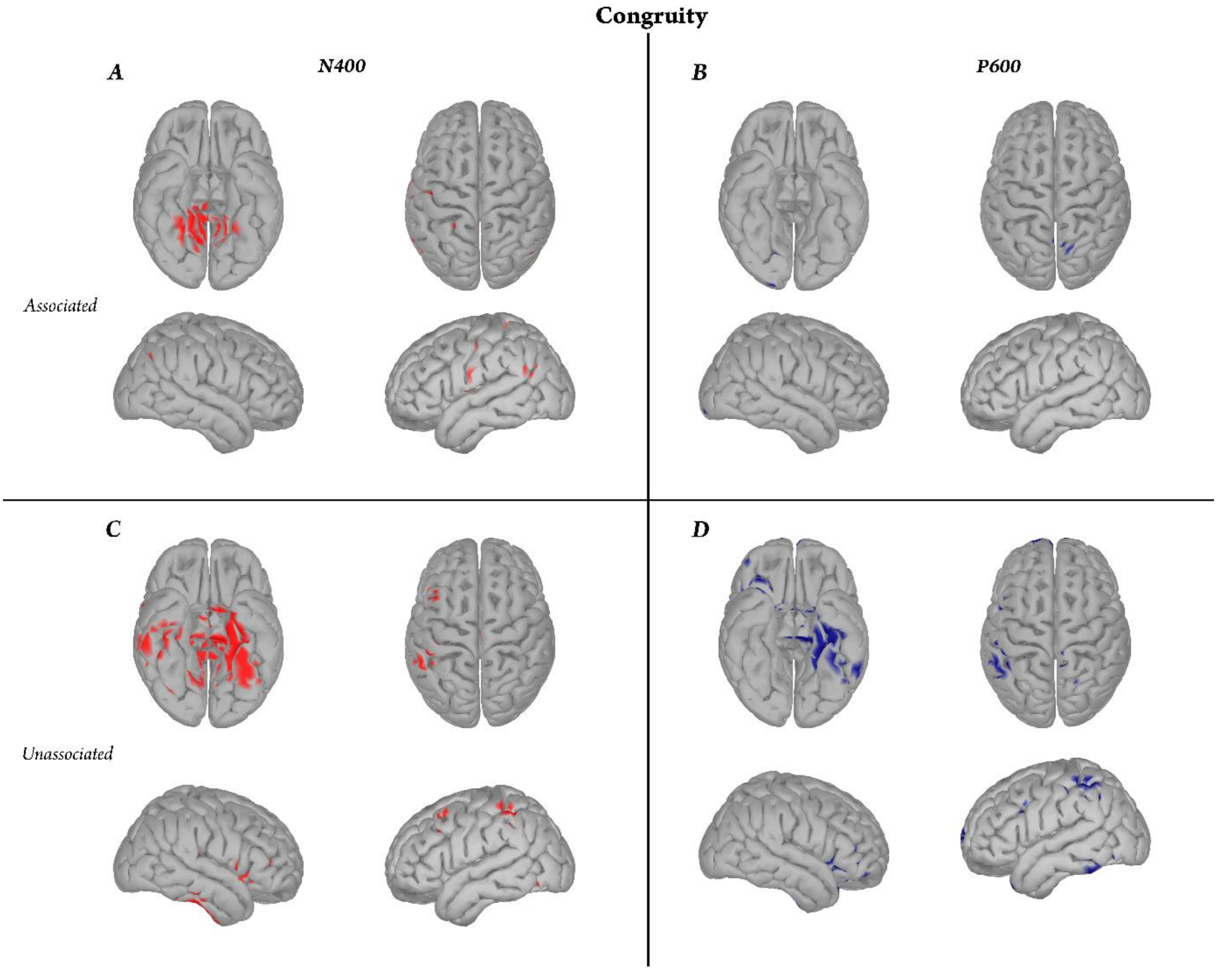

Fig. S1: Effect of congruity evaluated with source localization for sentences with and without associations in the time-windows of N400 (presented in red) and P600 (presented in blue) ERPs. The highlighted areas indicate the brain regions with statistically significant effect of congruity (p<0.05).

P600 ERP

For the P600 time window (500 – 800 ms), the *effect of congruity* (for overview, see Table S2 I.) was mainly significant on the left parahippocampal gyrus but for sentences without associations (Fig. S1 D, area >7cm^2, p=0.0061) only. This effect was additionally significant on left fusiform gyrus (area=5.26 cm^2, p=0.0071), left supramarginal gyrus (area=2.82 cm^2, p=0.0048) and frontal pole on left hemisphere (area=2.22cm^2, p=0.0054). The *effect of association* (Table S2 II. for overview), for incongruent sentences, was significant only in the left anterior inferior frontal cortex (Fig. S2, D, area around 1.65cm^2, p=0.008). For congruent sentences, this effect was present on (Fig. S2, B) the frontal poles bihemispherically (more on the right hemisphere with area= 6.55cm^2, p=0.0053) and the right superior parietal lobule (area=4.61cm^2, p=0.0054).

Table S2: Effects of congruity and association for P600 ERP.

	I. Congruity effect		II. Association effect
**Associated (HA)**	- Left superior parietal cortex (small)	**Congruent**	- Right superior parietal cortex<br>- Frontal poles on both hemispheres (more on right)
**Unassociated (LA)**	- Left parahippocampal gyrus<br>- Left Fusiform gyrus<br>- Left supramarginal gyrus<br>- Left frontal pole	**Incongruent**	- Left anterior inferior frontal cortex

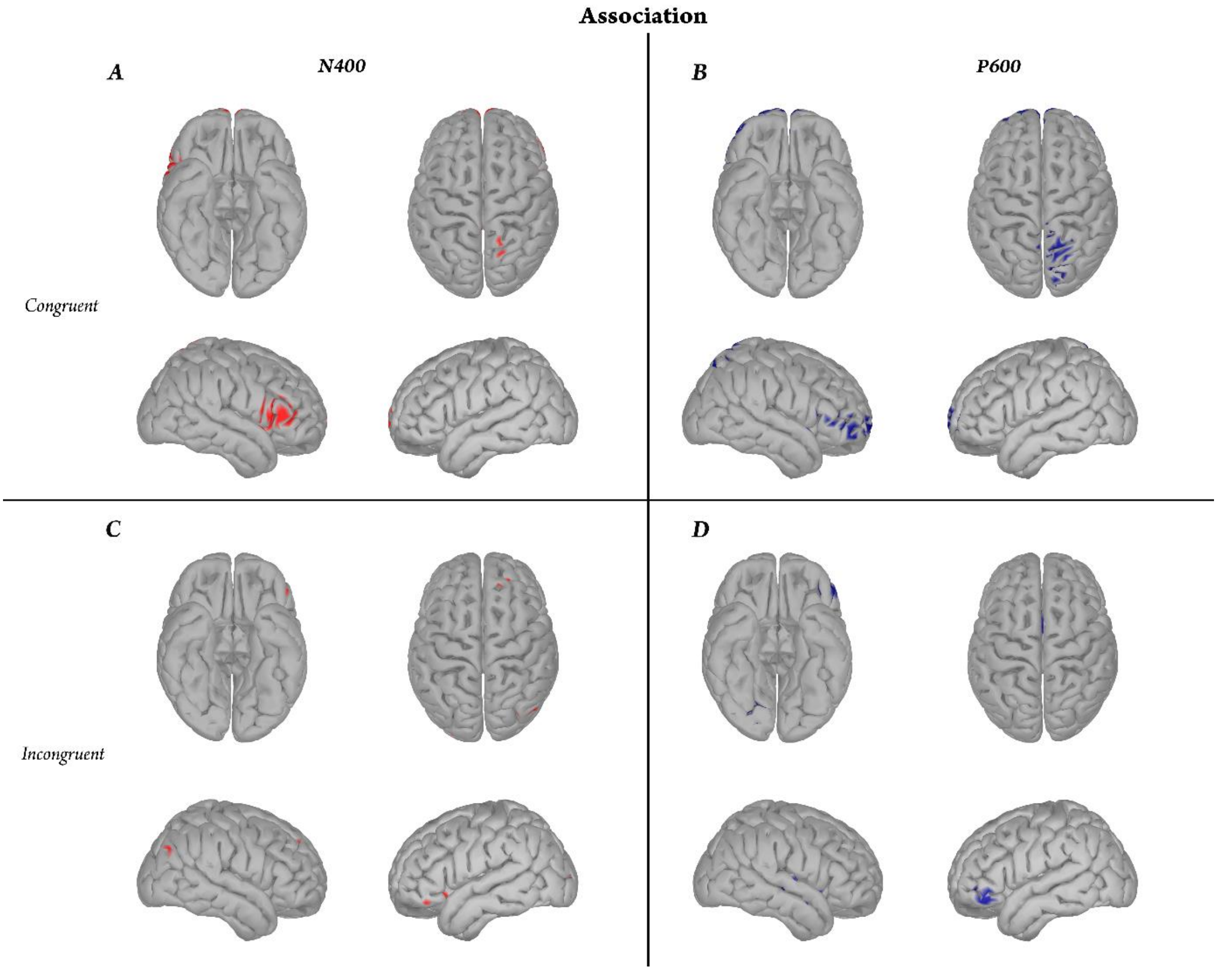

Fig. S2: Effect of association evaluated with source localization. The highlighted areas indicate the brain regions with statistically significant effect of association ($p<0.05$). Same conventions as in Fig. S1.

Activity of frontal poles during sentence processing

Our source localization analysis showed effects of congruity in the late time window in unassociated context and the effect of associations in congruent context in both time-windows in frontal poles. This indicates that these brain areas are involved in the re-analysis and the integration of the word in the active context. Indeed, as the prefrontal cortex participates in cognitive control[5], the re-analysis of the context, after the whole information becomes available, would be a logical function for these areas. We observed with our source localization analysis the effect of association in frontal poles bihemispherically in congruent context for both the early and late time windows, which we could not observe from our ERP analysis. Since in the N400 time-window, the temporal analysis of our ERP data did not reveal a significant difference between congruent associated (congHA) and unassociated (congLA) sentence groups, this observation cannot be regarded as an indicator of the difficulty of semantic retrieval. Since, the frontal poles were not associated to linguistic processing, but rather to more general cognitive processing and cognitive control [6]; it could explain the absence of this effect in the temporal analysis of our ERP data. The presence of this effect in the source localization could be explained by the participation of this brain region in task performance. Since in the behavioral data we also observed a lower performance for the congruent sentences that did not contain a probe word, it is possible that for congruent sentences, cognitive control was more involved in the performance of the task and the evaluation of the individual word, and, therefore, also the processing of intra-sentence associations. Additionally, this phenomenon is an indicator of the non-linearity of the inverse solution, according to which, the observed effects in source localization should not necessarily be observed in the ERP analysis.

References:

1. Becker, H. *et al.* Brains source imaging: from sparse to tensor models. *IEEE Signal Process. Mag.* 100–112 (2015). doi:10.1109/MSP.2015.2413711
2. Koles, Z. J. Trends in EEG source localization. *Electroencephalogr. Clin. Neurophysiol.* **106,** 127–137 (1998).

3. Gloor, P. Neuronal Generators and the Problem of Localization in Electroencephalography: Application of Volume Conductor Theory to Electroencephalography. *J. Clin. Neurophysiol.* **2,** 327–354 (1985).
4. Nunez, P. L. & Srinivasan, R. *Electric fields of the brain: The Neurophysics of EEG*. (2006).
5. Miller, E. K. THE PREFRONTAL CORTEX AND COGNITIVE CONTROL. *Nat. Rev. Neurosci.* **1,** 59–65 (2000).
6. Koechlin, E. Frontal pole function : What is specifically human ? *Trends Cogn. Sci.* **15,** 241 (2015).

APPENDIX 2: STIMULUS LIST

**Sentence stem**	**Target word**	**Sentence group**
De vaas met bloemen staat op de gelakte	tafel.	congHA
De secretaris typt de zakelijke	brief.	congHA
De gemeente verbood de sloop van het	gebouw.	congHA
De kameel lag te slapen op het goudkleurige	zand.	congHA
Na een uur nam de vrouw de kip uit de	oven.	congHA
De zieke soldaat ligt op een	bank.	congHA
De jagers schoten op het snelle	hert.	congHA
De groenteboer plukt de sappige	tomaten.	congHA
Het was een mistige en koude	ochtend.	congHA
De kogel veroorzaakte een pijnlijke	wonde.	congHA
Ze sliep rustig in haar warme	bed.	congHA
Een ketting breekt in haar zwakste	schakel.	congHA
Met een groot net ving de man veel	vis.	congHA
Er liggen twee ankers van een zwarte	boot.	congHA
De jongen borstelt de	hond.	congHA
Ik geef u een spuitje tegen de	verkoudheid.	congHA
De vruchten zijn rijp in de vroege	zomer.	congHA
De student heeft een kamer met bad en	douche.	congHA
De kapitein verhoogde de snelheid van zijn dure	schip.	congHA
De foto staat op de houten	kast.	congHA
De schildpad legt zijn eieren in een diepe	put.	congHA
De vader gaf zijn jarige dochter de	cadeaus.	congHA
Veel mensen verwarren een gewone verkoudheid met	griep.	congHA
De slome schapen strompelen achter de vermoeide	herder.	congHA
Hij begint een strijd tegen drugs en	alcohol.	congHA
Mijn oma breide de wollen	sokken.	congHA
De man wandelt met een bruine	loopstok.	congHA
Hij gooide het balletje naar de speelse	poes.	congHA
De kinderen schopten de bal in de	goal.	congHA
Jan kijkt naar het nieuws op	tv.	congHA
Sofie legde het bed op de	grond.	congHA
De vogel heeft een kenmerkende	snavel.	congHA
Ik gebruik de dikke jas voor de koude	winter.	congHA
Op de vloer ligt een dik	tapijt.	congHA
Iedere morgen koopt de man zijn vaste	krant.	congHA
De jongen bedankt de politieman voor de	hulp.	congHA
Mannen gaan weer gezonder	leven.	congHA

Allergie manifesteert zich met irritatie en	jeuk.	congHA
De jongen botste hard tegen de	muur.	congHA
De zakenman droeg een mooi	pak.	congHA
Het mooist verklede kind krijgt een	prijs.	congLA
De oude boeren verspreiden de stinkende	mest.	congLA
De toerist bestudeerde lang de	kaart.	congLA
Het meisje sprong over de brede	beek.	congLA
Gertie kocht erg dure sieraden in de	winkel.	congLA
Kaarsen geven de kamer een zachte warme	sfeer.	congLA
Een stagiair werkt eerst twee maanden op	proef.	congLA
De butler droeg het dienblad door de	zaal.	congLA
De padvinders beginnen met de lange	tocht.	congLA
Dit zijn de symbolen van reinheid en	kuisheid.	congLA
Hij vroeg de bisschop om	vergiffenis.	congLA
De jonge artsen namen de belangrijke	beslissing.	congLA
De verpleegster nam de helende	zalf.	congLA
Tom erfde grond van zijn	vader.	congLA
Er staat een lange rij voor het	loket.	congLA
De geur van het parfum vulde de hele	kamer.	congLA
De huisvrouw kookt een heerlijk	maal.	congLA
De vrouw kookt het fruit tot	moes.	congLA
Stijn kocht de ring voor zijn toekomstige	echtgenote.	congLA
Het is een wijn met een fraaie	smaak.	congLA
Ze herhaalde de korte	zin.	congLA
Ik ga meestal naar hetzelfde	restaurant.	congLA
De oude man heeft een zeer slecht	zicht.	congLA
Hij zag hun sombere	gezichten.	congLA
Elke dag opnieuw voert Walter een innerlijke	strijd.	congLA
Met mijn familie heb ik weinig	contact.	congLA
Omdat ze te laat kwam kreeg Marian een	straf.	congLA
Bij de aankomst bedanken de passagiers de	piloot.	congLA
Er zit een vliegje in mijn	soep.	congLA
Ze serveert de stamppot met aardappelen en bruine	bonen.	congLA
Els gaf hem haar juiste	nummer.	congLA
België heeft een zee als natuurlijke	grens.	congLA
Het zieke meisje omhelst haar	moeder.	congLA
Het kind is erg bang voor de	knufferbeer.	congLA
De meeste kittens zien ‘s nachts	niets.	congLA
We bewaren alle tekeningen in een	map.	congLA
Het pakje sigaretten zit in zijn	zak.	congLA
Soldaten doen gewoon hun	werk.	congLA
Sofie en Vincent waren eens een gelukkig	koppel.	congLA
De dief kwam het huis binnen via de	achterdeur.	congLA
De jongen bedankt de politieman voor de	agent.	incongHA
De dief kwam het huis binnen via de	baksteen.	incongHA
Ik ga meestal naar hetzelfde	gelijk.	incongHA

Het is een wijn met een fraaie	drank.	incongHA
De vruchten zijn rijp in de vroege	appel.	incongHA
De oude boeren verspreiden de stinkende	neus.	incongHA
De vaas met bloemen staat op de gelakte	nagels.	incongHA
De huisvrouw kookt heerlijke	keuken.	incongHA
Ze herhaalde de korte	rok.	incongHA
Elke dag opnieuw voert Walter een innerlijke	binnenkant.	incongHA
De meeste kittens zien ‘s nachts	donker.	incongHA
Omdat ze te laat kwam kreeg Marian een	verjaardag.	incongHA
Stijn kocht de ring voor zijn toekomstige	hoop.	incongHA
Sofie legde het bed op de	dromen.	incongHA
De jonge artsen namen de belangrijke	familie.	incongHA
Met mijn familie heb ik weinig	tekort.	incongHA
Mannen gaan weer gezonder	groenten.	incongHA
Jan kijkt naar het nieuws op	journaal.	incongHA
Els gaf hem haar juiste	fout.	incongHA
Ze serveert de stamppot met aardappelen en bruine	beer.	incongHA
De foto staat op de houten	boom.	incongHA
Tom erfde grond van zijn	aarde.	incongHA
Kaarsen geven de kamer een zachte warme	vuren.	incongHA
De kinderen schopten de bal in de	sport.	incongHA
Hij zag hun sombere	regen.	incongHA
Het meisje sprong over de brede	ruimte.	incongHA
Er staat een lange rij voor het	geduld.	incongHA
Het mooist verklede kind krijgt een	liefde.	incongHA
Het pakje sigaretten zit in zijn	zetel.	incongHA
Ik geef u een spuitje tegen de	dokter.	incongHA
Een stagiair werkt eerst twee maanden op	dag.	incongHA
De jagers schoten op het snelle	bos.	incongHA
De oude man heeft een zeer slecht	weer.	incongHA
De padvinders beginnen met de lange	giraf.	incongHA
De gemeente verbood de sloop van het	kussen.	incongHA
De man wandelt met een bruine	vakantie.	incongHA
Sofie en Vincent waren eens een gelukkige	lach.	incongHA
Gertie kocht erg dure sieraden in de	juwelen.	incongHA
De toerist bestudeerde lang de	studie.	incongHA
Hij begint een strijd tegen drugs en	film.	incongHA
De zieke soldaat ligt op een	bloem.	incongLA
Soldaten doen gewoon hun	sleutel.	incongLA
De kapitein verhoogde de snelheid van zijn dure	stoel.	incongLA
Na een uur nam de vrouw de kip uit de	trein.	incongLA
Hij vroeg de bisschop om	veer.	incongLA
We bewaren alle tekeningen in een	kool.	incongLA
De geur van het parfum vulde de hele	jongen.	incongLA
De vogel heeft een kenmerkende	wagen.	incongLA
Op de vloer ligt een dik	land.	incongLA

De kameel lag te slapen op het goudkleurige	sap.	incongLA
De verpleegster nam de helende	kassa.	incongLA
Ze sliep rustig in haar warme	bord.	incongLA
België heeft een zee als natuurlijke	broer.	incongLA
Een ketting breekt in haar zwakste	taart.	incongLA
Het was een mistige en koude	mislukking.	incongLA
Mijn oma breide de wollen	muntstuk.	incongLA
Veel mensen verwarren een gewone verkoudheid met	kaars.	incongLA
De slome schapen strompelen achter de vermoeide	broek.	incongLA
Hij gooide het balletje naar de speelse	afval.	incongLA
Dit zijn de symbolen van reinheid en	ober.	incongLA
Ik gebruik de dikke jas voor de koude	ring.	incongLA
Bij de aankomst bedanken de passagiers de	vork.	incongLA
De student heeft een kamer met bad en	vinger.	incongLA
De butler droeg het dienblad door de	lip.	incongLA
De jongen botste hard tegen de	hemel.	incongLA
De groenteboer plukt de sappige	naalden.	incongLA
De secretaris typt de zakelijke	lucht.	incongLA
De vrouw kookt het fruit tot	hemd.	incongLA
Allergie manifesteert zich met irritatie en	ijzer.	incongLA
De jongen borstelt de	ingang.	incongLA
De vader gaf zijn jarige dochter de	tuin.	incongLA
Er liggen twee ankers van een zwarte	ster.	incongLA
Met een groot net ving de man veel	vleugels.	incongLA
Het kind is erg bang voor de	boodschap.	incongLA
Het zieke meisje omhelst haar	paal.	incongLA
Er zit een vliegje in mijn	dier.	incongLA
De schildpad legt zijn eieren in een diepe	telefoon.	incongLA
Iedere morgen koopt de man zijn vaste	kooi.	incongLA
De zakenman droeg een mooi	doek.	incongLA
De kogel veroorzaakte een pijnlijke	bril.	incongLA
